# Case Series: Hyperbilirubinemia under elexacaftor/tezacaftor/ivacaftor in the presence of Gilbert’s syndrome

**DOI:** 10.1186/s12890-024-03114-6

**Published:** 2024-07-01

**Authors:** Julia Weitzel, Matthias Welsner, Christian Taube, Manfred Ballmann, Sivagurunathan Sutharsan

**Affiliations:** 1 Department of Pediatrics, Rostock University Medical Centre, Rostock, Germany; 2 Department of Pulmonary Medicine, Adult Cystic Fibrosis Center, University Hospital Essen - Ruhrlandklinik, Essen, North Rhine-Westphalia Germany

**Keywords:** Cystic fibrosis, Elexacaftor/tezacaftor/ivacaftor, Hyperbilirubinemia, Gilbert’s syndrome, Liver dysfunction, Drug intolerance

## Abstract

Liver-related side effects are a known complication of treatment with elexacaftor/tezacaftor/ivacaftor (ETI) for cystic fibrosis (CF). Gilbert’s syndrome is caused by a genetic mutation that reduces activity of the enzyme UDP glucuronosyltransferase 1 polypeptide A1 (UGT1A1), causing elevated levels of unconjugated bilirubin in the blood and duodenal bile. The presence of Gilbert’s syndrome and CF might represent additive risk factors for liver-related adverse events during ETI treatment. This case series describes six people with CF (pwCF) in whom previously unknown Gilbert’s syndrome was unmasked after initiation of treatment with ETI. Although all patients had some level of hepatic dysfunction and/or elevated levels of bilirubin after initiation of ETI, the clinical course varied. Only one patient had to stop ETI therapy altogether, while the others were able to continue treatment (some at a reduced dosage and others at the full recommended daily dosage). All patients, even those using a lower dosage, experienced clinical benefit during ETI therapy. Gilbert’s syndrome is not a contraindication for ETI therapy but may be mistaken for a risk factor for liver-related adverse events in pwCF. This is something that physicians need to be aware of in pwCF who show liver adverse events during ETI therapy.

## Introduction

The approval of the cystic fibrosis transmembrane conductance regulator (CFTR) modulator triple therapy elexacaftor/tezacaftor/ivacaftor (ETI) in 2019 was a breakthrough in the treatment of people with cystic fibrosis (pwCF). Therapy with ETI is approved for use in patients with at least one F508del allele, which includes about 85% of all pwCF [1–3]. Phase 3 trials [1, 2] and the recently published PROMISE study [3] have shown that treatment with ETI significantly improves the percent predicted forced expiratory volume in 1 s (FEV_1_), sweat chloride concentration and body mass index, and reduces the number of pulmonary exacerbations. In the PROMISE study, these improvements were greatest in patients naive to CFTR modulator therapy, but also detectable in those previously treated with single or double CFTR modulator therapy [3].

In the 445 − 102 Phase 3 study [2], adverse events in pwCF treated with ETI were of mild or moderate severity occurred in 33.2% and 50.5% of patients, respectively. Elevated aminotransferase levels occurred in 22 patients (10.9%); these elevations were > 3, >5, > 8 times the upper limit of normal (ULN) in 16 patients (7.9%), 5 patients (2.5%), and 3 patients (1.5%), respectively [2]. It was reported that no patient had an aminotransferase level > 3 times the ULN concurrent with a bilirubin level > 2 times the ULN during treatment with ETI [2]. There was no detailed information about any isolated increase in bilirubin levels or recommendations for medical conduct in these cases.

The prescribing information for ETI states that no dosage adjustment is required in patients with mildly impaired liver function. In those with moderate hepatic dysfunction treatment with ETI is not recommended but could be used if there is a clear medical indication for treatment under careful consideration and risk-benefit assessment.

The CFTR is present in the apical membrane of the bile duct epithelium and CFTR dysfunction leads to an increase in bile viscosity, which can result in obstruction of the small bile ducts with inflammatory sequelae and local fibrotic remodeling of the liver [5, 15]. Overall, pwCF are more likely to experience liver impairment of any severity compared with the general population [6, 7]. Furthermore, pwCF also experience chronic bile salt loss from the small intestine or bile salt malabsorption, which induces resorption of unconjugated bilirubin from the large intestine [8, 9].

Bilirubin is the final product of the breakdown of hemoglobin. While normal bilirubin levels are typically less than 1.0 mg/dl [10, 11], jaundice occurs when the serum bilirubin level exceeds 3 mg/dl [11]. Unconjugated bilirubin is released into the plasma and reversibly binds to albumin [10]. When the level of unconjugated bilirubin exceeds albumin binding capacity, unconjugated bilirubin will accumulate in fatty tissue such as the brain. Thus, it can cause neurological deficits in different extents especially in young children and babies with immature blood-brain-barrier and can even be fatal if the patient is a carrier of heterozygous Crigler-Najjar mutation or in concomitant hemolytic disorders, e.g. G-6-PD deficiency or thalassemia [9, 11]. Therefore, bilirubin needs to be converted into a form that can be eliminated from the body. Gilbert’s syndrome is caused by a homozygous genetic mutation of autosomal dominant or autosomal recessive inheritance or in some rare cases by heterozygous or mosaic mutation [4, 11] that reduces activity of the enzyme UDP glucuronosyltransferase. In Gilbert’s syndrome, principal bilirubin conjugation to indirect bilirubin is reduced to 30% of normal, causing elevated levels of unconjugated bilirubin in the blood and the duodenal bile [8]. Gilbert’s syndrome is common, affecting about 8% of population, with a higher incidence in men than women. The presence of Gilbert’s syndrome and CF might mislead to additive risk factors for liver-related adverse events during ETI treatment.

This case series describes six pwCF in whom previously unknown Gilbert’s syndrome was unmasked after initiation of treatment with ETI.

## Case description

Patient 1 [F508del/R553X, UGT1A1*28 7/7] presented with severe malaise and jaundice 2 weeks after initiation of treatment with ETI. Blood testing showed hyperbilirubinemia, but aspartate aminotransferase (AST) and alanine aminotransferase (ALT) levels were within the normal range. Treatment with ETI was temporarily paused. Although there was an outbreak of hepatitis A in town where the patient and their family lived, viral hepatitis was ruled out as a cause of the laboratory abnormalities. No other causes for elevated bilirubin levels could be detected, and abdominal ultrasound was normal. The patient’s father was found to have Gilbert’s syndrome. Therefore, the patient underwent genetic testing and was positive for UGT1A1. Over subsequent weeks, the patient experienced multiple flare-ups of hyperbilirubinemia due to upper respiratory tract infections and pulmonary exacerbations. Attempts to restart therapy on two separate occasions were unsuccessful. Then the patient and their parents revoked consent for ETI therapy.

Patient 2 [F508del/R553X, UGT1A1*28 7/7] had a direct relative with Gilbert’s syndrome and was therefore screened prior to starting ETI therapy; genetic testing showed that they were positive for UGT1A1. Based on experience with the direct relative Patient 1, the starting dose of ETI was reduced for Patient 2. Therapy was well tolerated, and follow-up appointments showed no signs of hyperbilirubinemia or increased AST/ALT. It was then possible to titrate ETI up to the recommended dosage without any signs of drug intolerance or other side effects.

The remaining patients were also all positive for UGT1A1. Patient 3 [F508del/F508del] and Patient 4 [F508del/F508del] showed a slight increase in bilirubin parameters at their 3-monthly check-ups, but no ETI dosage adjustment was needed. In Patient 5 [F508del/N1303K], the total bilirubin level was more than twice the normal value at the first 3-month follow-up after ETI therapy initiation. This necessitated a dosage reduction; the patient was able to continue treatment and showed good benefit at the next follow-up appointment.

Patient 6 [F508/1717-1G > A] had known CF hepatopathy, sonographic steatosis hepatis and cirrhosis. They showed bilirubin > 2 times the ULN at 3 months after therapy initiation, and the ETI dosage was reduced. At the 6-month follow-up, transaminase levels were increased but the bilirubin level remained < 2 times the ULN. There were no sonographic signs of cholestasis or cholangitis. They were classified as Child-Pugh score 5–6 and the increased transaminase level was determined to be related to CF hepatopathy rather than ETI intolerance because bilirubin levels were within normal limits.

**Table 1:** Detailed patient data

Patient (sex) Follow-up	Age, years	AST, U/L	ALT, U/L	Bilirubin, mg/dL	ppFEV_1_	Sweat chloride, mmol/L	Weight, kg	Mutations	Notes
1 (M)	16							F508del/R553X UGT1A1*28 7/7	Therapy restart at a lower dosage failed Consent for ETI therapy revoked
*ETI start*		39	22	2.6	77	88	57.2		
*2 weeks*		35	33	7.1		44	59		
*Pause (3 month)*		33	22	2.3	75	106	58.7		
*2 weeks*		37	26	6.5		89	59.8		
2 (F)	22							F508del/R553X UGT1A1*28 7/7	Therapy started at a reduced dosage Full dosage tolerated from week 3
*ETI start*		20	12	1.4	82	89	48		
*2 weeks*		20	13	1.3	107	38	48		
*9 months*		22	20	2.2	97		45.7		
3 (F)	44							F508del/F508del UGT1A1	No therapy adjustment needed
*ETI start*		6	21	1.6	60	105	52		
*3 months*		12	21	1.8	65	51	52		
*6 months*		9	25	2.5	67	48	56		
4 (M)	32							F508del/F508del UGT1A1	No therapy adjustment needed
*ETI start*		97	73	1.3	69	105	67		
*3 months*		79	103	2	74	60	71.3		
*6 months*		110	115	2.2	75	48	71.3		
5 (M)	32							F508del/N1303K UGT1A1	Reduced dosage used from the 3-months follow-up
*ETI start*		17	22	1.8	65	98	91		
*3 months*		21	27	2.7	90	82	89		
*6 months*		27	23	2.5	78	55	90		
6 (M)	20							F508/1717-1G > A UGT1A1	Reduced dose since 3-months follow-up Hepatic cirrhosis and hepatic steatosis Child-Pugh score 5–6
*ETI start*		31	31	2.1	96	98	72		
*3 months*		33	53	2.9	104	53	72		
*6 months*		286	165	2.2	99	48	72		

ALT, alanine aminotransferase; AST, aspartate aminotransferase; F, female; M, male; ppFEV_1_; forced expiratory volume in 1 s

ULN AST/ALT (M) 50/50 U/L, ULN AST/ALT (F) 35/35 U/L, ULN Bilirubin 1.1 mg/dL

## Discussion

Although Gilbert syndrome is common in the general population, it is rarely described in pwCF [11]. This case series shows very individual reactions and ability to tolerate ETI therapy in the presence of Gilbert Syndrome in pwCF.

About 40% of all pwCF develop hepatobiliary complications over time [12]. CFTR dysfunction leads to viscosity increase of the bile, which can cause obstruction of the small bile ducts with inflammatory sequelae and remodelling of the liver [5]. Patients can show hepatomegaly, persisting high levels of AST, ALT and/or gamma-glutamyl transferase, increased echogenicity on ultrasound as a sign of steatosis hepatis, heterogenous echogenicity of the liver or nodular tissue changes [7].

In case of hepatobiliary complications during treatment with ETI, it is important to rule out other differential diagnoses, such as infectious diseases (e.g. Epstein Barr virus, cytomegalovirus, hepatitis A, B or C virus, etc.) or autoimmune hepatitis. Furthermore, drug-drug interactions need to be considered and ruled out. If there are elevated levels of total and unconjugated bilirubin with normal transaminase levels it is important to think of Gilbert’s syndrome and perform genetic testing.

If Gilbert’s syndrome is diagnosed patients additionally need to be screened for interfering causes of increased levels of unconjugated bilirubin levels, such as fasting, dehydration, sleep deprivation, mental or physical stress, before considering drug-related hyperbilirubinemia [11].

All six pwCF in this series were positive for UGT1A1 mutation (see Table 1). Interfering causes of hyperbilirubinemia in Gilbert’s Syndrome were ruled out. Only Patient 1 showed severe symptoms of hyperbilirubinemia and exacerbation of Gilbert’s syndrome, which resulted in the discontinuation of therapy. There may even be differences between siblings in the ability to tolerate ETI therapy. In terms of clinical effectiveness, and regardless of the ETI dosage used, all patients in the current series showed improved lung function and reduced sweat chloride concentration (Table 1).

There are two studies with similar findings. In a North American three-center case series by Patel et al. found seven pwCF with elevated serum bilirubin after start of ETI therapy associated with Gilbert’s syndrome. In three pwCF elevated bilirubin values led to temporary suspension of ETI therapy. Therapy could be successfully resumed once the diagnosis of Gilbert’s syndrome was established in all three patients [13]. A previous Italian retrospective multi-center study screened 503 pwCF on ETI therapy, 52 (10,3%) developed hyperbilirubinemia of which 17 patients were positive for Gilbert’s syndrome (homozygous *n* = 16). Beforehand all patients were screened for other causes (infections or autoimmunity or hematological abnormalities), abdominal ultrasound showed CF–associated liver disease in three cases (cirrhosis *n* = 2 and hepatic steatosis *n* = 1). None of the Italian patients discontinued ETI treatment [14].

Although hyperbilirubinemia and increased liver enzyme levels are common side effects of ETI therapy, patients need to be closely monitored for additional signs of liver function impairment. This includes screening for unconjugated hyperbilirubinemia and if present, to check for Gilbert’s syndrome or other conjugation disorders. In some cases, the ETI dosage may need to be adjusted or treatment may need to be discontinued when there are signs of malaise and severe liver impairment. The treatment with ursodesoxycholic acid (UDAC) in pwCF and CF-related liver disease remains controversial and there are no studies or systemic reviews on how UDAC may affect Gilberts syndrome in pwCF and in general [12].

The current case series shows that Gilbert’s syndrome is not a contraindication for ETI therapy. Our study identified isolated elevations in serum total bilirubin among individuals with cystic fibrosis (pwCF) following initiation of elexacaftor/tezacaftor/ivacaftor (ETI), which unmasked Gilbert’s Syndrome. Longitudinal analysis demonstrated significant increases in total bilirubin levels after ETI initiation, without concurrent elevations in liver transaminases in affected individuals. Given the benign nature of elevated bilirubin levels in Gilbert’s Syndrome, ETI therapy could generally be continued without interruption in these patients, with one exception in our cohort. It is essential to consider genetic testing for Gilbert’s Syndrome in pwCF who develop isolated hyperbilirubinemia after starting ETI. It is noteworthy that isolated elevation of unconjugated bilirubin and total bilirubin could potentially lead to misunderstandings related to liver-related risks. In some pwCF, markedly elevated unconjugated bilirubin and total bilirubin levels may lead to jaundice, which can induce anxiety and affect therapy adherence. Jaundice in these patients manifests as yellowing of the skin and eyes, indicating elevated bilirubin levels in the bloodstream. This can cause discomfort and psychological distress, potentially impacting patient well-being and therapy compliance.

Accurate diagnosis can prevent unnecessary therapy discontinuation, ensuring continued access to a treatment that provides substantial health benefits in cystic fibrosis. Physicians should be vigilant regarding factors influencing therapy tolerance in these patients, underscoring the need for further research to fully elucidate the interaction between Gilbert syndrome and CFTR modulator therapy.

Therefore, pwCF on ETI therapy should:

Monitor bilirubin levels closely and interpret them in the context of Gilbert’s Syndrome.

Educate patients about the benign nature of Gilbert’s Syndrome and its implications for therapy continuation.

Address concerns related to jaundice promptly to mitigate anxiety and enhance therapy adherence.

This comprehensive approach aims to optimize therapy management and improve outcomes for pwCF receiving CFTR modulator therapy and concurrent Gilbert Syndrome.

## Data Availability

The dataset analysed in the case series is available from the corresponding author on reasonable request.

## Abbreviations

ALT: alanine aminotransferase
AST: aspartate aminotransferase
CF: cystic fibrosis
CFTR: cystic fibrosis transmembrane conductance regulator
ETI: elexacaftor/tezacaftor/ivacaftor
F: female
M: male
ppFEV_1_: forced expiratory volume in 1 s
pwCF: people with cystic fibrosis
UDAC: ursodesoxycholic acid
UDP: uridine diphosphate
UGT1A1: uridine diphosphate glucuronosyl-transferase 1 polypeptide A1
